# Thrombospondin-1: Multiple Paths to Inflammation

**DOI:** 10.1155/2011/296069

**Published:** 2011-07-03

**Authors:** Zenaida Lopez-Dee, Kenneth Pidcock, Linda S. Gutierrez

**Affiliations:** Department of Biology, Wilkes University, Wilkes-Barre, PA 18766, USA

## Abstract

Inflammation is a defensive process against tissue injury. Once this self-protective strategy is initiated, an effective resolution of the process is crucial to avoid major and unnecessary tissue damage. If the underlying event inducing inflammation is not addressed and homeostasis is not restored, this process can become chronic and lead to angiogenesis and carcinogenesis. Thrombospondin-1 (TSP-1) is a matricellular protein involved in angiogenesis, cancer, and inflammation. The effects of TSP-1 have been studied in many preclinical tumor models, and mimetic peptides are being tested in cancer clinical trials. However, the molecular mechanisms explaining its role in inflammatory processes are not well understood. This paper will discuss the role of TSP-1 in inflammation and its interaction with key receptors that may explain its functions in that process. Recent literature will be reviewed showing novel mechanisms by which this multifaceted protein could modulate the inflammatory process and impact its resolution.

## 1. Introduction

Thrombospondin-1 (TSP-1) is a matricellular glycoprotein first discovered in activated platelets. [1] TSP-1 is the best-studied member of the thrombospondin (TSP) family, which consists of five extracellular calcium-binding multifunctional proteins: TSP-1, TSP-2, TSP-3, TSP-4, and TSP-5. TSP-1 and TSP-2 are structurally similar, and they are expressed on the cell surface during physiological events. A variety of normal cells, including endothelial cells, fibroblasts, adipocytes, smooth muscle cells, monocytes, macrophages, and transformed cells such as malignant glioma cells, secrete TSP-1 [2, 3]. 

TSP-1 binds to protein components of the extracellular matrix, such as fibronectin. By this way, TSP-1 is stored in the extracellular matrix where it folds and changes its conformation. TSP-1-specific domains bind to proteoglycans, membrane proteins such as integrins, and other matrix proteins expressed by a variety of cells [4, 5]. 

**Figure 1:**
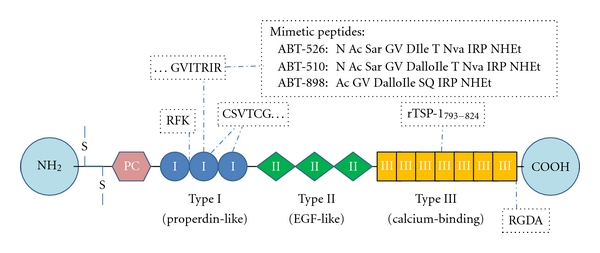
Schematic representation of the structure of TSP-1 and its receptor sites. TSP-1 is a large, homotrimeric molecule (420 kDa). Each monomer consists of an interchain disulfide bond (S=S), procollagen homology domain (PC), Type I, II, and III repeats. The amino and carboxyl terminals are globular [8]. RFK and RGDA are the binding sites for TGF*β* and the integrins, respectively. CSVTCG is the receptor site for CD36. The C-terminal domain of TSP-1 binds CD47. TSP-1 mimetic peptides have been designed from the heptapeptide GVITRIR of the second type-1 repeat. ABT-526 and ABT-510 are nonapeptides and enantiomers of each other, where D-Ile was replaced with D-allo-Ile in ABT-510 [33]. Sar, Nva, and NHEt are abbreviations for sarcosine, norvaline, and ethylamide. A second generation mimetic peptide, ABT-898, has been reported to have increased potency and slower clearance [95]. These peptides have been used in clinical studies for their antiangiogenic properties. Recombinant fragment rTSP1_793–824_ has been studied in experimental erosive arthritis [72].

TSP-1 contains an N-terminal globular domain that binds heparin, the type I, type II, and type III repeats, and a terminal globular domain. The structure of TSP-1 is schematically shown in Figure 1. The NH_2_-terminal, heparin-binding domain of TSP-1 interacts with low-density lipoprotein receptor-related protein (LRP1). LRP1 releases any metalloproteases already bounded to TSP-1, modulating the protease activity [6]. This TSP-1 domain also binds heparin sulfate proteoglycans and a number of integrins that have an important function in angiogenesis, chemotaxis adhesion, and cell motility [7]. All five members of the TSP family have the repeat domains type II and III, but only TSP-1 and TSP-2 contain the type I repeats [8]. Type I repeats, also called thrombospondin structural homology repeats (TSRs), inhibit angiogenesis by activating CD36 and inducing apoptosis in endothelial cells [9]. CD36 (also known as fatty acid translocase, FAT) is a glycosylated protein member of the class B scavenger receptor family. It plays an important role in multiple processes such as fatty acid and glucose metabolism. CD36 is found on the surface of diverse cell types and binds to many ligands, including TSP-1 [10, 11]. It has been reported that upon binding with TSP-1, CD36 dimerizes, becoming actively involved in signal transduction [12]. However, activation of CD36 as a monomer has also been reported [13]. The adhesive and antiangiogenic functions of TSP-1 have been mainly attributed to its interaction with CD36. Other domains of TSP-1 can, however, impact these functions by interacting with other key receptors as it will be discussed in succeeding sections.

TSP-1 is a major activator of transforming growth factor (TGF*β*1) [14]. Indeed, it is the only member of the thrombospondin family that activates TGF*β*1. TGF*β*1 mediates wound healing, cell proliferation, extracellular matrix formation, and the immune response. This multifunctional cytokine is secreted to the extracellular matrix in its inactive form, by virtue of its noncovalent association with the latency-associated peptide (LAP) [15]. The activating function of TSP-1 is due to the amino acid sequence RFK located in the TSR [14–16]. TSP-1 releases TGF*β*1 from its latent form when it interacts with the N-terminal region of LAP and binds the mature TGF*β*1. This interaction results in the formation of a complex that involves conformational changes in TGF*β*1, [16] making it accessible to its receptor. LAP is crucial for TGF*β*1 activation and regulates many of its functions; additionally, LAP has functions in inflammation independently of TGF*β*1, such as the induction of chemotaxis of monocytes to injured tissues [17]. 

The type 3 repeats of TSP-1 are calcium-binding domains. They contain amino acid sequences that interact with the neutrophil elastase, and upon this binding these repeats activate neutrophils [18, 19]. These type 3 repeats also inhibit the binding of fibroblast growth factor to endothelial cells, reducing angiogenesis [20].

The COOH-terminal domain of TSP-1 binds to CD47, also known as integrin-associated protein. [21] This domain also interacts with integrins such as *β*1 and *β*v6 integrins and actively binds to proteoglycans allowing cell adhesion and spreading [7]. These and other interactions significantly affect angiogenesis, cell proliferation, and immune responses. TSP-1 binding with CD47 also regulates nitric oxide (NO), a biogas, quite important in both normal and pathological events [22]. By modulating the effects of NO, the carboxy-terminal domain of TSP-1 has important function in vasodilation and chemotaxis [22]. CD47 impacts angiogenesis to a large extent. This receptor inhibits NO as well as all its vascular functions even when TSP-1 is present at very low (physiological) concentrations [22]. Analysis of wound bed vascularity at 72 hours after skin grafting from TSP-1 and CD47 null mice shows significant increased numbers of blood vessels [23]. Most recently it has been reported that CD47 associates with the receptor of vascular endothelial growth factor, VEGFR2 [24]. However, the binding of CD47 with TSP1 or other ligands inhibits VEGFR2 phosphorylation and further angiogenesis.

This paper focuses on well-known interactions of TSP-1 with key receptors and growth factors during the initial inflammatory events throughout the chronic inflammatory processes. New developments are also herein discussed, showing the involvement of TSP-1 in pivotal transcriptional pathways related to inflammation and inflammation-induced carcinogenesis.

## 2. TSP-1 in Acute Inflammation 

Inflammation is the reaction of living tissue to local injury. The inflammatory acute process begins when cells sense the injury, and they release chemical mediators called cytokines. Local macrophages express surface membrane receptors called toll-like receptors (TLR) that recognize specific types of antigens. Once activated, TLR triggers the release of more cytokines promoting inflammation and attracting white blood cells. Cytokines will promote leukocytosis by inducing factors favoring the rapid release of neutrophils from the red bone marrow. Neutrophils enter the blood stream, and by diapedesis they emigrate outside the blood vessels.

The vascular system is a key factor in acute inflammation. Vasodilation increases the capillary permeability and promotes exudate formation. The slow blood flow favors the release of chemical mediators and enhances local edema. Chemotactic agents accelerate the migration of leukocytes to the site of injury such as monocytes, which later become macrophages, engulfing any on-site cell debris or pathogens. In addition, mast cells (producing histamine), injured tissue cells, phagocytes, lymphocytes, basophils, and blood proteins are all sources of inflammatory mediators. 

TSP-1 is transiently released early during the acute phase of inflammation, and multiple factors seem to modulate the release of TSP-1 during this process. TSP-1 is strongly expressed in neutrophils, inducing an intense chemotactic response to injured tissues [2]. TSP-1 is secreted in response to inflammation, promoting the resolution of the inflammatory process and facilitating phagocytosis of damaged cells [25, 26]. Thus, enhanced production of TSP-1 could be a compensatory mechanism for controlling the immune response and protecting tissues from excessive damage. 

**Figure 2:**
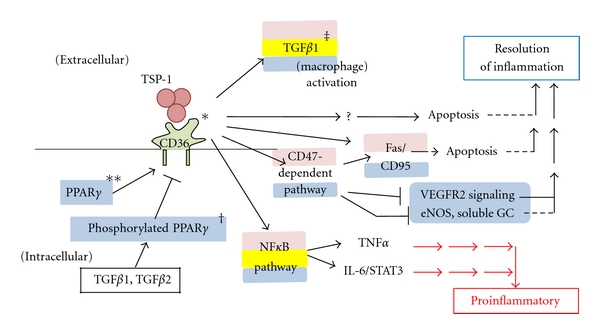
Dual role of CD-36—TSP-1 interaction in inflammation. Expression of CD36 is positively regulated by PPAR*γ* and negatively regulated by TGF*β*. As an integral membrane protein, CD36 binds many ligands including TSP-1. The CD36-TSP-1 interaction involves conformational changes in TSP-1. This interaction mediates apoptotic effects via the CD47-dependent pathway, which has multiple effects. The CD36-TSP-1 interaction can also activate macrophage TGF*β*1 and the NF*κ*B pathway *[98, 99], **[100–102], ^†^[100], ^‡^[103]. Blue shading indicates pathways occurring in endothelial cells, pink shading in macrophages, and yellow shading in epithelial cells.

TSP-1 mediates macrophage phagocytosis of apoptotic cells via CD36. This receptor is coexpressed with TSP-1 in macrophages and endothelial cells, and, by binding with CD36 (Figure 2), TSP-1 induces apoptosis in endothelial cells [9]. By activating CD36, TSP-1 also controls blood flow and leukocyte infiltration modulating the action of the NO pathway in injured tissues [27]. NO is a gas produced when L-arginine is converted to L-citruline by the enzyme nitric oxide synthase (NOS). There are four different isoforms of NOS, neuronal (nNOS), endothelial (eNOS), mitochondrial (mtNOS), and the inducible isoform (iNOS). The first two are secreted during normal physiological events, but only iNOS is expressed upon inflammatory stimuli [28].

The effects of NO in inflammation have been extensively recognized in a variety of studies. NO can modulate leukocyte adhesion in a dose-dependent manner [27]. At low doses, NO is anti-inflammatory and antiangiogenic but, after inflammatory stimuli, high levels of NO are secreted promoting angiogenesis and leukocyte adhesion to the endothelium [29]. TSP-1 could inhibit the soluble guanylyl cyclase system in endothelial cells and consequently the activation of NO by interacting with CD36 and CD47 [22]. Through this mechanism, TSP-1 inhibits inflammation by blocking adhesion and activation of leukocytes to the endothelium and diminishing angiogenesis [22, 30, 31]. 

Another factor interacting with TSP-1 during early inflammation is the peroxisome proliferator-activated receptor (PPAR). PPAR is a member of the nuclear hormone receptor superfamily of transcription factors. When PPAR is absent in leukocytes, the leukocytes secrete high levels of TSP-1 [32]. PPAR*γ* greatly enhances the proapoptotic effects of the TSP-1-derived peptide ABT510 (Abbott Laboratories) [33, 34]. This peptide corresponds to the TSR of TSP-1 and induces vascular apoptosis *in vitro* and *in vivo* through its interaction with CD36 [35]. By using a PPAR*γ* agonist, the expression of CD36 in endothelial cells is enhanced, improving the antiangiogenic effects of ABT510 in a CD36-dependent manner [34].

The TSP-1 receptor CD47 is critical for the migration of leukocytes through endothelial and epithelial barriers [36]. CD47 is strongly expressed in polymorphonuclear cells, and its activation enhances the expression of TSP-1 in leukocytes [37]. TSP-1 also induces leukocytic apoptosis through the CD47 pathway. CD47 can directly cause apoptosis through mitochondrial mechanisms, or by activation of the Fas/CD95 pathway [38]. Expression of CD47 in apoptotic granulocytes can influence the phagocytic functions of the macrophages in inflammatory sites suggesting a critical role of this factor in the resolution of the process [39] (Figure 2).

## 3. TSP-1 in Chronic Inflammation and Adaptive Immunity

Acute inflammation could advance to a resolution, progress to the formation of an abscess, walling off by fibrotic capsule, or evolve as scar upon tissue destruction, fibrin and collagen deposition. In many instances, this process continues as chronic inflammation. Chronic inflammation is characterized by infiltration of mononuclear cells, macrophages, lymphocytes, and plasma cells. Chronically inflamed tissues have fibroblast proliferation, angiogenesis, tissue destruction, and fibrosis. 

Monocytes and macrophages are key elements of chronic inflammation. They invade the injured area during the acute process but, if the cause is not eliminated, infiltration by macrophages persists for long periods of time. The continued secretion of chemotactic factors allows the constant supply of monocytes from the blood and their conversion to macrophages. These cells are key for further lymphocyte infiltration, fibroblast proliferation, tissue destruction, and fibrosis. Lymphocytes arise from the hemoblasts of the bone marrow, and later they develop immunocompentence and self-tolerance. T lymphocytes mature in the thymus and confer cell-mediated immunity. Plasma cells or B lymphocytes produce antibodies against antigens persisting in the area and therefore provide humoral immunity. They become immunocompetent when antigen-specific receptors appear on their surface. Plasmatic cells and macrophages are called antigen-presenting cells (APCs). Included in this group are dendritic cells (DCs), which internalize antigens and present antigenic determinants on their surface for recognition by T lymphocytes. They are part of the adaptive immune system that recognizes something as foreign and acts to immobilize and remove it. During the early stages of injury and inflammation, high levels of TSP-1 increase the tolerance of DC to antigens, ending the inflammatory response. TSP-1 can modulate inflammation by inhibiting or enhancing the secretion of the cytokine interleukin 10 (IL10), by this way, TSP-1 can also regulate the functions of DC [26]. In addition, after adding IL-6, IL-10, or TGF*β*1 to cultured DC, they become immune tolerant and show upregulation of intracellular TSP-1 [40]. TSP-1 also inhibits the function of APC by suppressing their capacity to sensitize T-cells in the host. This is demonstrated in a corneal transplantation model, in which most of the corneal TSP-1 null allografts are rejected [41]. CD47 has also a crucial role in T-cell activation [42, 43]. Interaction of TSP-1 with CD47 promotes the activation of thymus-derived CD4+ CD25+ T regulatory cells (Tregs). Through this mechanism, CD47 helps to maintain self-tolerance inducing a suppressive phenotype [25]. 

 It has been recently reported that bacterial pathogenesis may be mediated by CD47. Suppression of CD47 or TSP-1 expression in DC by using small interfering RNA (siRNA) technique actually protects newborn mice against bacterial (*Escherichia coli*) meningitis [44]. Again, the loss of CD47 activity prevents the maturation of the DCs and the production of inflammatory cytokines [44]. In conclusion, CD47 seems to have pivotal functions in inflammation and provides a major mechanistic pathway for the functions of TSP-1 in that process. 

Finally, the deficiency of CD36 enhances the severity of bacterial and malaria infection. Cd36^−/−^ mice exhibit an impaired early proinflammatory response to infection, elevation of cytokines, and higher mortality [45, 46]. These findings suggest that CD36 is quite critical for the recognition and clearance of pathogens in acute and chronic infections. By binding to this receptor, TSP-1 could modulate the inflammatory process by activating macrophages and favoring phagocytosis. During chronic inflammation, these adaptive immune mechanisms provide defense against disease and are regulated by cellular interactions and cytokines. B lymphocytes secrete antibodies that bind to infectious agents and label them for destruction or elimination. Once inside a cell, a pathogen becomes inaccessible to those antibodies and cytotoxic T cells could destroy them by inducing apoptosis of the cell host. Regulatory T cells can modulate the secretion of cytokines enhancing the functions of macrophages and B-lymphocytes. TSP-1 has been reported to decrease immune responses by inhibition of T-cell effectors, or by directly inducing T cell apoptosis [47, 48]. In addition, by binding to *α*4*β*1 integrin TSP-1 promotes T-cell adhesion and chemotaxis [43].

**Figure 3:**
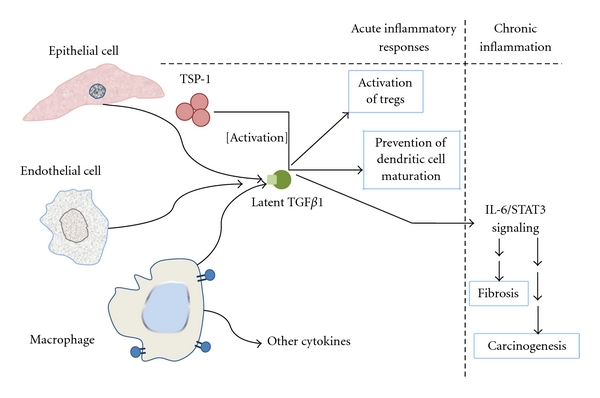
The homotrimeric TSP-1 activates latent TGF*β*1 by binding the N-terminal propeptide LAP and the mature TGF*β*1. The binding results in conformational changes in TGF*β*1 and allows TGF*β*1 to be recognized by its receptor. Mature, active TGF*β*1 has been reported to decrease dendritic cell maturation and activate T cells. An opposing role of TGF*β*1 results in fibrosis.

 TGF*β*1 activation is a crucial element in intestinal homeostasis [49]. In the intestinal tract, an abnormal response to the normal gut flora is a characteristic of the pathogenesis of inflammatory bowel disease (IBD). Mucosal T cells from patients with IBD express high levels of Smad7, an inhibitor of TGF*β*1 signaling. By this mechanism, TGF*β*1 mediates intestinal healing and susceptibility to injury [50–53]. However, by activating TGF*β*1, TSP-1 also enhances fibrosis and compromises organ function [54, 55] (Figure 3). During the immune response, leukocytes produce reactive oxygen species (ROS) that include free radicals and peroxides. ROS are quite important for the killing of pathogens, but they can also produce cell damage. TGF*β*1 favors the formation of ROS, and, as a cycle, ROS can also activate TGF*β*1 promoting apoptosis and fibrosis [56].

 Angiogenesis is an active component of chronic inflammatory diseases such as rheumatoid arthritis, atherosclerosis, diabetic retinopathy, airway inflammation, and others [57]. TSP-1 exerts a powerful antiangiogenic effect, and this function has a significant impact in chronic inflammation. Activated endothelial cells secrete cytokines, chemokines, matrix metalloproteinases, and growth factors that can greatly influence the inflammatory reaction and promote carcinogenesis.

## 4. TSP-1 in Inflammatory Diseases and Animal Models 

TSP-1 deficient mice display extensive acute pneumonia, leukocytosis, pancreatitis, and inflammatory infiltrates in the lacrimal glands [26, 58, 59]. This phenotype suggests that TSP-1 has a significant role in inflammation, a role that has not been exhaustively analyzed. TSP-1 has anti-inflammatory and proinflammatory effects observed in several diseases and animal models. These contrasting functions in inflammation are possible due to interactions with multiple receptors or to the presence of specific matricellular proteins in injured tissue. In addition, TSP-1 might act by a biphasic or dose-dependent mechanism. 

TSP-1 enhances fibrosis and renal damage by its interaction with TGF*β*1 [54], while LSKL, a peptide antagonist of TSP-1, reduces renal interstitial fibrosis in a rat experimental model of kidney disease [60]. This effect is attributed to the competitive properties of LSKL that prevents TSP-1-mediated activation of TGF*β*1 [60]. 

TSP-1 is also expressed in glomerulopathies and is considered an early marker of inflammation and fibrosis [61]. The development of diabetic nephropathy is attenuated in TSP-1-deficient mice as demonstrated by a significant reduction of glomerulosclerosis, glomerular matrix accumulation, podocyte injury, renal infiltration with inflammatory cells, and alterations of renal functional parameters [62]. 

As an activator of TGF*β*1, TSP-1 could modulate the functions of TGF*β*1 in cardiovascular diseases, atherosclerosis, and obesity. Inflammatory cells secrete TSP-1 during the acute phase of the healing process in myocardial infarction. Infarcted murine hearts show marked upregulation of TGF*β*1. In addition, TSP-1 is selectively expressed in the infarcted border suggesting that TSP-1 might inhibit the expansion of the inflammation by activating TGF*β*1 [63]. 

In the inflammatory processes leading to atherosclerosis, TSP-1 deficiency enhances inflammation by accelerating plaque maturation and necrosis in ApoE-deficient mice. ApoE^−/−^TSP-1^−/−^ mice also show enhance expression of metalloproteinase 9. A defective phagocytosis appears to accelerate necrosis favoring additional inflammation and macrophage infiltration [64]. However, TSP-1 expression is increased in response to high glucose in the wall of large vessels and accelerates atherosclerosis and other pathological events observed in diabetes [65]. 

TSP-1 mRNA is significantly associated with obesity and insulin resistance in nondiabetic patients. This correlation is explained by the TSP-1-dependent TGF*β*1 activation that leads to upregulation of plasminogen activator inhibitor 1 (PAI-1) gene expression. Elevated circulating PAI-1 levels are detected in insulin resistance and metabolic syndrome [66]. 

Mice lacking TSP-1 or the integrin *β*6 subunit (TSP-1^−/−^ and Itgb6^−/−^ mice, resp.) develop inflammation with a phenotype similar to that seen in TGF*β*1 null mice. The inflammation is not as severe as the one found in Tgfb1^−/−^ mice, which develop marked infiltrates of activated T cells in multiple organs and die soon after weaning [51]. 

TSP-1 null mice have a more severe course of acute induced colitis; they display increased bleeding and colonic inflammation compared to WT mice controls [67]. TSP-1 deficiency significantly enhances angiogenesis and dysplasia when the disease is cyclically induced. TSP-1-deficient mice under multiple cycles of 2.5% of dextran sodium sulfate for induction of colitis die before the cycles are completed. These mice show high incidence of megacolon and peritonitis due to the deeper infiltration of leukocytes into the muscularis and intestinal perforation. In order to increase survival, doses need to be significantly reduced [68]. The use of the TSP-mimetic peptide ABT510 drastically reduces intestinal inflammation and angiogenesis and enhances the expression of CD36, which suggests that CD36 modulates inflammation in this model [67]. Additionally, the interaction of TSP-1 with the NO pathway seems to be involved in the antiangiogenic mechanisms mediated by the ABT510 in cancers [31, 69], and it might also explain the anti-inflammatory effects of this peptide in the colitis model [70]. Similarly, in a rat model with bleomycin-induced lung injury, a CD36 peptide also decreases inflammation and fibrosis in the lungs [71]. 

A synthetic peptide derived from the TSP-1 type 3 repeats (Figure 1) also diminishes inflammation and angiogenesis in an experimental model of erosive arthritis [72]. This peptide decreases angiogenesis, leukocyte infiltration, and thickening of the synovial lining of the joint. In the spleen and liver, this peptide significantly reduces the formation of granulomas [73]. However, TSP-1 is upregulated in monocytes, and tissues from patients with rheumatoid arthritis (RA) [74, 75] and high plasmatic levels of TSP-1 are correlated with proinflammatory cytokines in these patients [76]. 

TSP-1 also exerts proinflammatory effects in certain types of myositis [77, 78]. The chemotactic effect of TSP-1 in leukocytes by its binding to CD47 perpetuates the muscle inflammation in response to high levels of TNF-alpha.

In transplantation research, most of the corneal TSP-1 null allografts are rejected, suggesting a protolerogenic function [41, 79]. Interestingly, when TSP-1 is inhibited by siRNA-transfection in pancreatic islet cells, improved revascularization and function are observed in the pancreatic grafts. Antiangiogenic compounds, such as TSP-1, might be useful for achieving a better perfusion and oxygenation in organ transplants [80].

## 5. TSP-1 and Transcriptional Pathways Leading to Inflammation-Induced Carcinogenesis

### 5.1. The Nuclear Factor Kappa-Light-Chain-Enhancer of Activated B Cells (NF-*κ*B)

The NF-*κ*B is a transcription factor present in almost all animal cell types. It is involved in many biological processes such as inflammation, immunity, differentiation, cell growth, tumorigenesis, and apoptosis. Normally NF-*κ*B is maintained in the cytoplasm by its inhibitor (I*κ*B). Phosphorylation and subsequent degradation of I*κ*B releases NF-*κ*B and allows it to be translocated to the nucleus. Once inside the nucleus, this transcription factor induces its target genes, thereby inhibiting apoptosis, promoting inflammation, angiogenesis, and carcinogenesis [81]. Importantly, NF-*κ*B activates the transcription of genes such as TNF-alpha and IL-6, which have major roles in regulating the immune response [82].

Recently, it has been reported that blocking the activation of NF-*κ*B upregulates TSP-1 expression in rat granulation tissue [83]. TSP-1 has a major role in wound healing, and a significant delay of the healing process is observed in TSP-1^−/−^ mice [84].

The link between TSP-1 and NF-*κ*B seems to be closely related to mechanisms of angiogenesis and carcinogenesis. TSP-1 enhances the binding of NF-*κ*B to DNA inhibiting angiogenesis, and these events are reverted by blocking the NF-*κ*B pathway [85]. Activation of NF-*κ*B by oxidized low-density lipoprotein (oxLDL), a specific ligand of CD36, is reduced in macrophages of patients with CD36 deficiency [86]. TSP-1 might activate this pathway by its interaction with this receptor. 

Studies in prostate cancers indicate that the combined decrease of NF-*κ*B and increase of TSP-1, modulated by the expression of the androgen receptor, exert antitumor effects [87]. The TSP-1-derived peptide angiocidin has antitumor effects and induces the differentiation of monocytes to macrophages by activating the NF-*κ*B pathway [88].

### 5.2. The Signal Transducer and Activator of Transcription 3 (STAT3)

In response to cytokines and growth factors, members of the STAT family are phosphorylated by receptor-associated kinases forming homo- or heterodimers. These factors translocate to the nucleus where they act as transcription activators. The lack of STAT3, the most important member of this family, actually leads to embryonic lethality [89]. STAT3 mediates the expression of a variety of genes in response to cell stimuli and thus plays a key role in many cellular processes such as cell growth and apoptosis. The binding of IL-6 to the gp130 receptor triggers the phosphorylation of STAT3 by JAK2 [90, 91]. 

It has been reported that downregulation of TGF*β*1 in epithelial cells inhibits the secretion of IL-6. By this mechanism, blocking TGF*β*1 inhibits the IL-6/STAT3 signaling preventing colorectal cancer [92]. Furthermore, activated STAT3 has been recently reported as crucial for intestinal carcinogenesis in colitis-associated cancer [93]. In addition, protein profiling of head and neck squamous carcinomas shows a significant decrease of several proteins such TSP-1 and TGF*β*1 with concomitant increase of STAT3 [94].

In a model of angiogenesis, higher levels of IL-6 are secreted by TSP-1^−/−^ aortic explants, and similar results are found in a mouse model of colitis [95]. The higher levels of IL-6 are lowered upon activation of TGF*β*1 only in WT aortas. The activity of IL-6 was specifically examined through the STAT3 pathway in colonic tissues evaluating the status of the phosphorylated forms of STAT3 (p-STAT3). Interestingly, p-STAT3 expression is highly expressed in TSP-1^−/−^ colons and almost abolished in colons from mice treated with a new TSP-1-mimetic ABT-898 (Abbott Laboratories) [96]. These data suggest that this mimetic peptide directly binds to TSP-1 receptors such as CD36 or indirectly interacts with other membrane domains to downregulate STAT3 pathway, thereby depressing the immune response [95].

 The expression of cytokines such as IL-6 and TNF*α* is also regulated by NF-*κ*B. Therefore, activation of this pathway is critical for further activation of STAT3 in colitis [97]. These data indicate a cross talk between NF-*κ*B and STAT3 in inflammation and cancer. Therefore, linking TSP-1- mediated mechanisms to these fundamental transcriptional pathways warrants further investigation.

## 6. Conclusion

TSP-1 interacts with a variety of factors in a synergistic way, playing a crucial role in many stages of the inflammatory response. We discussed herein the interactions of TSP-1 with well-known partners such as CD36, CD47, NO, and TGF*β*1. We also reviewed recent developments explaining how TSP-1 and its derived peptides directly or indirectly regulate inflammatory events in animal models and human diseases. 

TSP-1 is generally found elevated in inflammatory processes and in several instances is proinflammatory. Leukocytes migrate to areas of injury or infection in elevated numbers; similarly, TSP-1 is secreted at high levels in some areas in order to activate specific mechanisms for regulating the inflammatory response. In some cases, TSP-1 activates leukocytes, enhances chemotaxis, and accelerates fibrosis. In other instances, TSP-1 prevents injury and progressive damage and enhances homeostasis. 

Finally, new insights about key signaling pathways such as NF-*κ*B and STAT3 were discussed. Because chronic inflammation is linked with tumor development, further studies analyzing the role of TSP-1 in these pathways could elucidate the similar contradictory functions of TSP-1 in cancer. The unraveling of these mechanisms will make possible the development of new and more effective therapies for controlling inflammation and blocking carcinogenesis.
